# Compensation Stability and Workforce Retention During COVID-19: A Paired Comparative Study of Home Care Nurses

**DOI:** 10.3390/healthcare14030414

**Published:** 2026-02-06

**Authors:** Naudrey Parker-Jenkins, John Kwame Duah

**Affiliations:** 1 Doctor of Health Administration Program, College of Health Professions, Central Michigan University, Mount Pleasant, MI 48859, USA; 2 Health Services Administration Program, Department of Political Science, Auburn University, Auburn, AL 36849, USA

**Keywords:** home care nursing, workforce retention, COVID-19, compensation satisfaction, paired comparative study, health system preparedness

## Abstract

**Highlights:**

**What are the main findings?**

- Compensation satisfaction among home care nurses remained stable before and during COVID-19.
- Selected retention-related perceptions, particularly financial stability and feeling well paid, changed significantly and were shaped by organizational and preparedness-related factors, including leadership, communication, and professional growth opportunities.

**What are the implications of the main findings?**

- Compensation stability by itself does not guarantee workforce retention during public health emergencies. While it acts as a hygiene factor, true retention relies on motivators like strong leadership and opportunities for professional growth.
- Health system preparedness strategies must integrate leadership, professional growth, and workforce resilience planning.

**Abstract:**

**Background/Objectives:** Home care nurses play a vital role in maintaining continuity of care for vulnerable populations. However, the COVID-19 pandemic exposed long-standing vulnerabilities in the workforce within home and community-based services. While compensation is often emphasized as a primary driver of workforce retention, less is known about how compensation satisfaction and retention intentions changed over time during a public health emergency. **Methods**: This study employed a cross-sectional survey with retrospective paired comparisons among home care nurses at five home care agencies in Maryland. To assess temporal changes, respondents retrospectively evaluated compensation satisfaction, job satisfaction, and retention intentions before and during the COVID-19 pandemic. Paired samples *t*-tests were used to examine within-respondent differences across time periods. Herzberg’s Motivator-Hygiene Theory guided the interpretation of these changes in extrinsic and intrinsic workplace factors. **Results**: Compensation satisfaction did not differ significantly between the pre-pandemic and pandemic periods. In contrast, selected retention-related perceptions, particularly financial stability and feeling well paid, changed significantly and were associated with organizational and preparedness-related factors such as leadership, communication, and professional growth opportunities. Several intrinsic job satisfaction dimensions improved during the pandemic, while resource-related challenges remained. **Conclusions**: The findings suggest that compensation stability functioned as a hygiene factor that was insufficient to secure workforce retention during the COVID-19 pandemic. Retention intentions were shaped by the interaction of financial security and organizational preparedness. Workforce policies to strengthen home care systems should combine compensation strategies with leadership development, professional growth opportunities, and emergency preparedness planning to build resilience during future crises.

## 1. Introduction

Home care nurses play vital roles in providing essential health services to older adults, people with chronic conditions, and medically vulnerable groups in home and community settings. Unlike hospital care, home care takes place in decentralized environments that often lack immediate clinical backup, consistent access to personal protective equipment, and standardized emergency preparedness infrastructure [1,2,3]. Despite their centrality in continuity across life stages, home care nurses have historically received less policy attention than hospital-based clinicians, especially in workforce planning and emergency preparedness initiatives [4].

Building on these long-standing challenges, the COVID-19 pandemic brought the realities of home care work into sharper focus. As hospitals restricted admissions and long-term care facilities faced outbreaks, demand for home-based services rose substantially, placing additional strain on an already fragile workforce [5]. During this period, home care nurses navigated increased occupational risk, rapidly shifting care protocols, staffing shortages, and heightened emotional and physical demands. These pressures shaped day-to-day practice and raised broader questions about job satisfaction and workforce stability [6,7]. Taken together, these conditions underscore the need to understand how workforce experiences in home care settings evolved during the pandemic and how organizational responses influenced nurses’ intentions to remain in the field.

### 1.1. Compensation, Job Satisfaction, and Retention in Home Care Settings

Compensation has long been acknowledged as a central factor shaping recruitment and retention in nursing and allied health professions. In home care settings, comparatively lower wages, limited benefits, and fewer opportunities for advancement relative to institutional care have been consistently linked to high turnover rates [8,9,10]. Prior research further shows that inadequate compensation contributes to workforce instability, particularly among nurses and direct care workers who provide long-term services in community-based environments [11]. At the same time, evidence indicates that compensation alone does not fully determine retention decisions. Organizational factors, including leadership quality, supervisory support, communication, professional development opportunities, and perceived respect, shape job satisfaction and intentions to remain employed [12,13,14]. During crisis conditions, these non-financial factors may become even more salient as health care workers navigate uncertainty, safety concerns, and rapidly evolving expectations [15].

Against this backdrop, the COVID-19 pandemic offers a critical context for examining how compensation satisfaction and organizational conditions relate to retention. While many health care workers experienced heightened stress and burnout, some organizations implemented adaptive strategies such as increased leadership visibility, improved communication, and professional recognition that may have mitigated workforce attrition despite limited changes in pay structures [16]. Examining how these dynamics shifted before and during the pandemic among home care nurses can inform workforce policy and preparedness planning.

### 1.2. Public Health Emergencies, Preparedness, and Workforce Stability

Public health emergencies place extraordinary demands on health systems and their workforces, often revealing gaps in preparedness, coordination, and resource allocation. The COVID-19 pandemic served as a global stress test, showing that workforce resilience depends on financial compensation, organizational readiness, leadership capacity, and supportive infrastructure [17]. Studies in diverse healthcare settings have shown that inadequate preparedness can heighten workforce stress, undermine morale, and increase turnover risk during emergencies [18]. Furthermore, health systems research has highlighted the significance of contextual and organizational factors, including leadership, communication, and resource mobilization, in shaping workforce experiences during pandemics [19].

In their examination of COVID-19 preparedness and response dynamics, Duah et al. emphasized that gaps in infrastructure and workforce capacity can compromise system performance and amplify strain on frontline personnel during public health emergencies [20]. Although much of the preparedness literature has focused on hospitals and border health systems, similar dynamics are highly relevant to home and community-based care settings, which play essential roles in surge capacity and continuity of care during crises. Clarifying the role of preparedness conditions in home care settings can help refine strategies aimed at supporting workforce stability during future public health emergencies [17,18,19,20].

### 1.3. Herzberg’s Motivator-Hygiene Theory

This study is guided by Herzberg’s Motivator-Hygiene Theory, which distinguishes between extrinsic hygiene factors, such as salary, job security, and working conditions, and intrinsic motivator factors, including professional growth, recognition, responsibility, and meaningful work [21]. According to the theory, hygiene factors help prevent dissatisfaction but are insufficient on their own to sustain motivation or long-term retention. Motivator factors, in contrast, are more closely linked to job satisfaction and continued engagement.

Herzberg’s framework is especially relevant for understanding workforce experiences during crisis conditions. In public health emergencies, compensation stability may serve as a baseline requirement that reduces dissatisfaction, while organizational and professional conditions influence employees’ decisions to remain in their roles. In this study, compensation, job security, and benefits were conceptualized as hygiene factors, whereas leadership, collaboration, communication, decision-making involvement, and professional growth were treated as motivator factors. Applying this framework provides a structured way to interpret changes in compensation satisfaction and retention intentions among home care nurses before and during the COVID-19 pandemic.

### 1.4. Study Aims, Research Question, and Hypotheses

This study had three primary aims:

1. To compare compensation satisfaction among home care nurses before and during the COVID-19 pandemic.
2. To examine temporal differences in retention intentions across the same period.
3. To interpret these differences through a health policy and preparedness approach informed by Herzberg’s Motivator-Hygiene Theory.

Guided by these aims, the study addressed the following primary research question:

How did compensation satisfaction and retention intentions among home care nurses differ before and during the COVID-19 pandemic, and what organizational factors were associated with these temporal differences?

Based on Herzberg’s Motivator-Hygiene Theory and prior workforce literature, the study evaluated the following hypotheses:

**H1.**  *Compensation satisfaction among home care nurses did not differ significantly between the pre-pandemic and pandemic periods.*

**H2.**  *Retention intentions among home care nurses differed significantly between the pre-pandemic and pandemic periods.*

**H3.**  *Organizational and preparedness-related factors were significantly associated with retention intentions during the pandemic period.*

## 2. Materials and Methods

### 2.1. Study Design

This study employed a cross-sectional survey with retrospective paired comparisons to examine perceived changes in compensation satisfaction and workforce retention intentions among home care nurses before and during the COVID-19 pandemic. Respondents were surveyed at a single point in time and asked to retrospectively assess selected job-related experiences across two reference periods: the pre-pandemic period and the pandemic period. This design enabled within-respondent comparisons of perceptions across time, minimized between-subject variability and respondent burden, and aligned with the study’s focus on temporal differences in experiences rather than causal inference. This retrospective, cross-sectional design supported an exploratory, descriptive analysis of perceived temporal differences in workforce experiences rather than a causal evaluation of specific interventions.

### 2.2. Study Setting and Participants

The study was conducted among home care nurses employed at five home care agencies in Silver Spring, Maryland. Data collection occurred between April and May 2023. Eligible participants included licensed nurses providing direct patient care in home and community-based settings during the COVID-19 pandemic. Nurses were eligible to participate if they were employed by one of the participating agencies during both the pre-pandemic and pandemic periods and were able to retrospectively evaluate their work experiences across these timeframes.

### 2.3. Data Collection and Survey Instrument

Data were collected through a structured, self-administered survey capturing demographic and employment characteristics, compensation satisfaction, job satisfaction, and retention intentions. To assess changes over time, respondents retrospectively rated their experiences during two reference periods, before and during the COVID-19 pandemic, using parallel survey items. Compensation satisfaction was measured using Likert-type items assessing perceptions of pay adequacy and financial stability. Retention intentions were assessed using items that captured respondents’ likelihood of remaining in their current position or in the home care workforce. Additional job satisfaction and organizational preparedness dimensions included perceptions of leadership support, communication, professional growth opportunities, and safety and preparedness practices. The survey instrument was informed by prior nursing and health workforce studies, and domains were constructed to reflect commonly examined extrinsic (hygiene) and intrinsic (motivator) workplace factors.

### 2.4. Statistical Analysis

Descriptive statistics summarized participant characteristics and key study variables. Paired samples *t*-tests examined within-respondent differences in compensation satisfaction, job satisfaction dimensions, and retention intentions between the pre-pandemic and pandemic periods, with statistical significance set at a two-tailed alpha of 0.05. Given the exploratory nature of this study and the modest sample size, no formal adjustment for multiple comparisons was applied. Instead, confidence intervals are reported to aid interpretation of the magnitude and precision of observed differences. All analyses were conducted in IBM SPSS Statistics version 28.

### 2.5. Ethical Considerations

This study was reviewed and approved by the Institutional Review Board of Central Michigan University (IRB Protocol #2022-1333) and classified as minimal risk research. The study was initially approved under an expedited review process, and a subsequent amendment addressing compensation satisfaction and job retention during the COVID-19 pandemic was also approved prior to data collection.

No direct personal identifiers were collected. The survey included demographic and employment-related characteristics. However, all responses were collected anonymously and could not be linked to individual participants. The data were stored securely, accessible only to the research team, and analyzed in aggregate form to protect participant confidentiality.

This journal manuscript is derived from and serves as a supplement to the first author’s doctoral dissertation research and is reported in accordance with the original Institutional Review Board approval.

### 2.6. Data Availability

The data supporting the findings of this study are not publicly available due to privacy and ethical restrictions protecting participant confidentiality. However, data may be available from the corresponding author upon reasonable request and with institutional approval.

## 3. Results

### 3.1. Sample Characteristics

Table 1 presents the demographic characteristics of the study sample. The respondents were predominantly female (83.0%) and African American (76.6%). The largest age group was 35–44 years (27.7%), followed by the 55–65 years age group (23.4%).

### 3.2. Compensation Satisfaction Before and During COVID-19

A paired-samples *t*-test assessed changes in compensation satisfaction before and during the COVID-19 pandemic. Mean differences for all compensation elements were close to zero, and their 95% confidence intervals included zero, indicating no meaningful shift in perceived compensation. There were no statistically significant differences in satisfaction with any compensation elements between the two periods (all *p* > 0.05; see Table 2).

### 3.3. Job Satisfaction Before and During COVID-19

Paired-samples *t*-tests were also conducted to evaluate changes in job satisfaction dimensions before and during the COVID-19 pandemic. Collaboration and leadership ratings were significantly higher during the pandemic (negative mean differences), whereas decision making, communication, and safety protocol ratings were significantly higher before the pandemic (positive mean differences), and in these cases, the positive mean differences indicate a decline in these dimensions during the pandemic rather than an improvement. The full details are presented in Table 3.

### 3.4. Changes in Retention Intentions Before and During the COVID-19 Pandemic

Paired-samples *t*-tests were employed to examine within-respondent differences in retention intentions before and during the COVID-19 pandemic. Retention-related factors assessed included perceptions of financial stability, job security, and being well paid. Descriptive statistics and inferential test results are presented in Table 4. Mean differences indicated statistically significant changes in selected retention-related factors across the two periods, with perceptions of financial stability and being well paid significantly higher during the pandemic (negative mean differences; *p* < 0.05). In addition, perceptions of professional growth also differed significantly between the two periods, underscoring the importance of developmental opportunities as a retention-related factor (see Table 4). In contrast, perceived job security did not show a statistically significant change over time (*p* > 0.05). Together, these results indicate that changes in retention intentions were not uniform across domains, with financial, compensation-related, and professional growth perceptions showing greater temporal variation than job security.

## 4. Discussion

### 4.1. Summary of Key Findings

Compensation satisfaction remained stable before and during the COVID-19 pandemic, with no significant differences across compensation domains. Some job satisfaction areas, especially collaboration and leadership, improved significantly during the pandemic, while others showed no meaningful change. Retention intentions also varied by domain: perceptions of financial stability and feeling well paid changed significantly, whereas perceived job security remained stable. These findings highlight domain-specific patterns and the tension between steady compensation satisfaction and changing perceptions of financial security among home care nurses during the pandemic.

### 4.2. Compensation Stability and Retention During a Public Health Emergency

A key finding of this study was that compensation satisfaction among home care nurses remained stable before and during the COVID-19 pandemic. Even amid widespread health system disruption, respondents reported no significant changes in satisfaction with salary, benefits, paid time off, or bonuses. This pattern aligns with previous research suggesting that compensation in home and community-based care is slow to change, even in times of crisis [1,4,9]. However, during a public health emergency of this scale, the lack of improvement in perceived compensation may reflect a missed opportunity for agencies and policymakers to recognize the increased risk, workload, and emotional burden faced by home care nurses. Notably, stable compensation did not mean stable retention intentions. Perceptions related to retention, especially financial stability and feeling adequately compensated, shifted significantly during the pandemic. This highlights a key distinction between steady compensation satisfaction and broader perceptions of economic security during public health crises. Prior studies have found similar patterns, where unchanging wages fail to buffer workers from increased uncertainty, workload, and risk during emergencies [6,15,16]. Taken together, these findings suggest that compensation stability may serve as a baseline condition that prevents dissatisfaction but does not, on its own, ensure workforce retention under emergency conditions. From a Herzbergian perspective, compensation may have acted mainly as a hygiene factor, while changes in financial security and organizational conditions influenced retention intentions. This interpretation aligns with long-standing workforce research indicating that retention decisions in nursing and direct care are shaped by multiple factors [8,13].

### 4.3. Organizational and Job Satisfaction Factors Shaping Workforce Experiences

Beyond compensation, this study identified meaningful changes in selected job satisfaction dimensions during the pandemic period. For example, improvements in collaboration, leadership, decision-making, communication, and safety protocols suggest that certain organizational responses helped to buffer the adverse effects of crisis conditions. These findings align with past evidence showing that leadership visibility, clear communication, and collaborative practice environments are central to sustaining workforce resilience during periods of system strain [7,12,19].

At the same time, not all job satisfaction dimensions improved. Ongoing challenges related to staffing, burnout prevention, and access to essential resources remained evident, reflecting structural constraints within the home care sector that predate the pandemic [1,10,11]. These mixed patterns underscore that organizational adaptations during COVID-19 were uneven and highly context-dependent, shaped by existing infrastructure, leadership capacity, and resource availability.

The observed improvements in selected intrinsic workplace factors are noteworthy because they indicate that even without substantial changes in compensation, organizational practices can help influence workforce experiences. Past research has similarly shown that supportive leadership and professional recognition can help offset some of the stressors associated with high-demand care environments, especially during emergencies [15,18].

### 4.4. Retention Intentions, Preparedness, and Workforce Stability

Retention intentions among home care nurses varied across domains. Significant changes were observed in perceptions of financial stability and being well paid, while job security remained relatively stable. This pattern highlights the complex ways public health emergencies reshape workforce decision-making. Continued employment may reflect labor market constraints or professional commitment, whereas shifting perceptions of financial adequacy may signal underlying vulnerability in workforce stability [9,11]. The preparedness literature has long shown that workforce resilience depends on both individual commitment and system-level readiness, including staffing flexibility, resource mobilization, and organizational support mechanisms [17,19]. Inadequate preparedness can amplify stress and uncertainty, thereby influencing retention intentions even when employment itself remains secure.

The findings from this study align with evidence that home health and hospice services faced significant operational and preparedness challenges during the COVID-19 pandemic [5]. In addition, broader analyses of peripheral and frontline health settings highlight similar gaps in system readiness and support [20]. Strengthening preparedness in these settings requires attention to workforce needs that extend beyond wages, including communication structures, leadership capacity, and mechanisms to support professional growth during crises.

### 4.5. Interpretation Through Herzberg’s Motivator-Hygiene Theory

Herzberg’s Motivator-Hygiene Theory provides a useful framework for interpreting the observed patterns in compensation satisfaction and retention intentions. In line with the theory, extrinsic hygiene factors such as salary and benefits appeared to prevent dissatisfaction but were insufficient to sustain motivation or retention during the pandemic period [21]. Compensation stability alone did not shield nurses from heightened uncertainty or fully shape their broader perceptions of financial security.

In contrast, changes observed in intrinsic motivator factors, including leadership, collaboration, and decision-making, were associated with improved job satisfaction during the pandemic. These findings support Herzberg’s assertion that motivator factors play a central role in fostering engagement and commitment, especially under challenging conditions [21].

**Figure 1:**
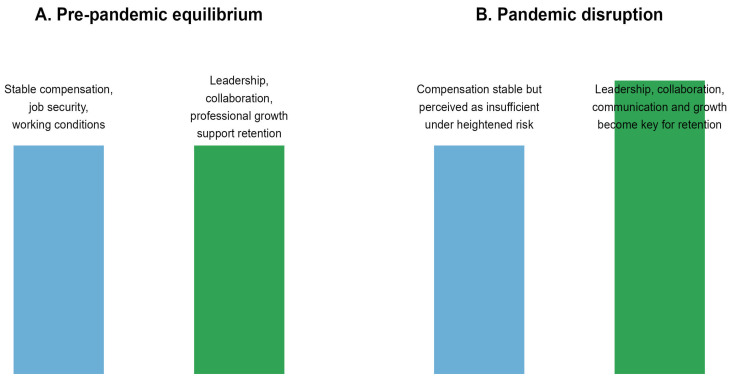
Conceptual application of Herzberg’s Motivator-Hygiene Theory to home care nurses’ retention during COVID-19. Note: Panel (**A**) illustrates the pre-pandemic equilibrium, where hygiene factors such as compensation, job security, and working conditions, along with motivator factors like leadership, collaboration, and professional growth, are in relative balance. This balance supports job satisfaction and retention. Panel (**B**) shows the pandemic disruption. Although hygiene factors remain stable, increased risk and uncertainty shift the main drivers of retention intentions to motivator factors and perceived financial security, even as compensation remains unchanged.

Applying this framework underscores the importance of integrated workforce strategies that address both hygiene and motivator factors. During public health emergencies, maintaining compensation stability may be necessary, yet it does not fully support retention on its own. Organizational practices that enhance professional meaning, recognition, and participation appear equally critical for sustaining the workforce during crisis conditions. Figure 1 summarizes this application of Herzberg’s Motivator-Hygiene Theory, contrasting pre-pandemic equilibrium with pandemic disruption in hygiene and motivator factors.

### 4.6. Policy and Practice Implications

The findings of this study have several implications for health policy and workforce planning in home and community-based care. First, policies that focus narrowly on wage stabilization without addressing organizational preparedness may fall short of supporting workforce retention during crises. State and federal initiatives aimed at improving direct care wages should be complemented by investments in leadership development, communication infrastructure, and workforce support systems [10,11]. Second, preparedness planning should explicitly incorporate home care settings as integral components of health system surge capacity and emergency preparedness. The COVID-19 pandemic has shown that home care nurses play a vital role in maintaining continuity of care for vulnerable populations, yet often do so without the institutional supports afforded to hospital-based clinicians [5,17]. Finally, workforce resilience strategies should prioritize professional growth opportunities and supportive organizational cultures that can buffer the psychological and operational strain associated with emergencies. Such approaches align with evidence from nursing and health workforce research underscoring the role of organizational context in shaping retention outcomes [12,13].

### 4.7. Strengths, Limitations, and Future Research Directions

This study offers several strengths, including the use of within-respondent paired comparisons, which enabled direct assessment of temporal changes in workforce perceptions. Focusing on home care nurses addresses an important gap in the literature, as much pandemic-related workforce research has centered on hospital settings. Nevertheless, the findings should be interpreted with certain considerations in mind. First, the study relied on retrospective self-reports, which may be subject to recall bias. Second, the sample was drawn from a specific geographic area, which may limit the generalizability of the findings to other settings. Although the sample size was modest, the paired comparison design increases statistical power by reducing between-subject variability, supporting its appropriateness for examining temporal differences.

Third, while the study captured multiple dimensions of job satisfaction and retention intentions, it did not examine long-term employment outcomes beyond the study period. These considerations should inform interpretation of the findings, but do not detract from the study’s contribution to understanding workforce experiences during the COVID-19 pandemic. Future research should assess how organizational adaptations implemented during the COVID-19 pandemic influence longer-term retention and workforce stability in home care settings. Mixed methods approaches or repeated assessments over time could provide deeper insight into how nurses interpret and respond to preparedness strategies. Expanding this line of inquiry across diverse geographic and policy contexts would further inform workforce planning and preparedness efforts.

## 5. Conclusions

This study clarifies how compensation satisfaction and retention intentions shifted for home care nurses during the COVID-19 pandemic, contributing to the understanding of health workforce stability. Drawing on a paired comparative approach and Herzberg’s Motivator-Hygiene Theory, the results show that stable compensation alone did not ensure workforce retention in a public health emergency. Retention was influenced by factors beyond pay, such as financial security, organizational readiness, leadership, and professional advancement opportunities.

The results reveal the limits of focusing solely on wages to strengthen the home care workforce. While compensation is essential, it cannot substitute for strong organizational infrastructure, effective communication, leadership support, or preparedness planning, all crucial for workforce resilience, especially in decentralized settings that serve vulnerable populations. The study emphasizes that workforce strategies should integrate home care into emergency preparedness and recognize compensation as a hygiene factor, best supported by motivators like leadership and professional growth.

These strategies are vital for both pandemic responses and sustaining long-term health system capacity as reliance on home-based care grows. In short, supporting a stable home care workforce during crises requires addressing the interplay of compensation, workplace conditions, and emergency readiness. Policies must reflect these complexities to maintain workforce continuity when it matters most.

## Figures and Tables

**Table 1:** Demographic characteristics of respondents.

Characteristic	Category	n	Percentage (%)
Gender	Male	8	17.0
	Female	39	83.0
Ethnicity	African American	36	76.58
	White	6	12.77
	Asian	2	4.26
	Multiracial	2	4.26
	Other	1	2.13
Age Group (years)	25–35	6	12.77
	35–44	13	27.66
	45–55	7	14.89
	55–65	11	23.40
Annual Income (USD)	≥65	10	21.28
	<$39,999	4	9.09
	$60,000–$79,999	9	20.45
	$80,000–$99,999	10	22.73
	$100,000–$119,999	7	15.91
	$120,000–$139,999	7	15.91
	≥$140,000	7	15.91

Note: Percentages are rounded to two decimal places, and percentages for annual income are based on valid responses only (n = 44).

**Table 2:** Paired samples *t*-tests for compensation satisfaction before and during COVID-19.

Paired Samples Test								
Difference (Before Minus During COVID-19)	Paired Differences					*t*	df	Significance
	Mean	Std. Deviation	Std. Error Mean	95% Confidence Interval of the Difference				*p*-Value (Two-Tailed)
				Lower	Upper			
Pair 1: Competitive Salary	0	1.251	0.182	−0.367	0.367	0	46	1
Pair 2: Satisf. with Stay Bonus	−0.149	0.955	0.139	−0.429	0.131	−1.069	46	0.291
Pair 3: Satisf. Paid Time Off	0.064	1.725	0.252	−0.443	0.57	0.254	46	0.801
Pair 4: Satisf. w. Shift Bonuses	−0.149	1.083	0.158	−0.467	0.169	−0.943	46	0.351
Pair 5: Flexible Schedule	0.234	1.165	0.17	−0.108	0.576	1.377	46	0.175
Pair 6: Satisf. w. Health Benefits	0.17	1.007	0.147	−0.125	0.466	1.159	46	0.252
Pair 7: Promotion Opportunities	0.213	1.178	0.172	−0.133	0.559	1.238	46	0.222

Note: Values represent paired samples *t*-tests comparing pre-pandemic and pandemic periods (before minus during COVID-19). Positive mean differences indicate higher satisfaction before the pandemic. Statistical significance was evaluated at *p* < 0.05 (two-tailed).

**Table 3:** Paired samples *t*-tests for job satisfaction dimensions before and during COVID-19.

Paired Samples Test								
Difference (Before Minus During COVID-19)	Paired Differences					*t*	df	Significance
	Mean	Std. Deviation	Std. Error Mean	95% Confidence Interval of the Difference				*p*-Value (Two-Tailed)
				Lower	Upper			
Supervision	0.444	2.546	0.379	−0.320	1.209	1.171	44	0.248
Collaboration	0.933	2.060	0.307	0.314	1.552	3.039	44	0.004
Staffing	−0.578	2.824	0.421	−1.426	0.271	−1.372	44	0.177
Leadership	1.089	2.204	0.328	0.427	1.751	3.315	44	0.002
Decision Making	0.911	2.162	0.322	0.262	1.561	2.827	44	0.007
Communication	0.911	2.420	0.361	0.184	1.638	2.526	44	0.015
Safety Protocol	0.909	2.390	0.360	0.182	1.636	2.523	44	0.015
PPE	0.578	2.684	0.400	−0.229	1.384	1.444	44	0.156
Absenteeism	0.044	2.067	0.308	−0.576	0.665	0.144	44	0.886
Burnout Prevention	−0.156	2.486	0.371	−0.902	0.591	−0.420	44	0.677
Infection Prevention	0.378	2.579	0.384	−0.397	1.152	0.983	44	0.331

Note: Mean differences represent before COVID-19 minus during COVID-19. Positive values indicate higher pre-pandemic ratings and negative values indicate improvement during the pandemic.

**Table 4:** Changes in retention-related perceptions before and during the COVID-19 pandemic.

Difference (Before Minus During COVID-19)	Paired Differences					*t*	df	Significance
	Mean	Std. Deviation	Std. Error Mean	95% Confidence Interval of the Difference				*p*-Value (Two-Tailed)
				Lower	Upper			
Resources/Assignments	−0.537	1.468	0.229	−1.000	−0.073	−2.341	40	0.024
Supervisor Appreciation	−0.024	1.423	0.220	−0.467	0.420	−0.108	41	0.914
Professional Growth	−0.488	1.362	0.213	−0.918	−0.058	−2.293	40	0.027
Job Availability	−0.381	1.834	0.283	−0.952	0.191	−1.346	41	0.186
Financial Stability	−0.524	1.174	0.181	−0.890	−0.158	−2.892	41	0.006
Job Security	−0.190	0.773	0.119	−0.431	0.050	−1.598	41	0.118
Well-Paid Job	−0.405	1.106	0.171	−0.749	−0.060	−2.373	41	0.022

Note: Paired samples *t*-tests were used to compare responses before and during the COVID-19 pandemic. Positive mean differences reflect higher ratings in the pre-pandemic period. Statistical significance was evaluated at a two-tailed α = 0.05.

## Data Availability

The data supporting this study are not publicly available due to privacy and ethical restrictions protecting participant confidentiality. Data may be obtained from the corresponding authors upon reasonable request and with institutional approval.

## Abbreviations

The following abbreviations are used in this manuscript:

CMS	Centers for Medicare & Medicaid Services
COVID-19	Coronavirus Disease 2019
HCBS	Home and Community-Based Services
IRB	Institutional Review Board
PPE	Personal Protective Equipment
